# Cortical thickness in Parkinson's disease: a coordinate-based meta-analysis

**DOI:** 10.18632/aging.202368

**Published:** 2021-01-10

**Authors:** LiQin Sheng, PanWen Zhao, HaiRong Ma, Joaquim Radua, ZhongQuan Yi, YuanYuan Shi, JianGuo Zhong, ZhenYu Dai, PingLei Pan

**Affiliations:** 1 Department of Neurology, Kunshan Hospital of Traditional Chinese Medicine, Kunshan, PR China; 2 Department of Central Laboratory, The Yancheng School of Clinical Medicine of Nanjing Medical University, Yancheng, PR China; 3 Imaging of Mood- and Anxiety-Related Disorders (IMARD) Group, Institut d’Investigacions Biomèdiques August Pi i Sunyer (IDIBAPS), CIBERSAM, Barcelona, Spain; 4 Early Psychosis: Interventions and Clinical-Detection (EPIC) Laboratory, Department of Psychosis Studies, Institute of Psychiatry, Psychology and Neuroscience, King’s College London, London, UK; 5 Centre for Psychiatric Research and Education, Department of Clinical Neuroscience, Karolinska Institutet, Stockholm, Sweden; 6 Department of Neurology, The Yancheng School of Clinical Medicine of Nanjing Medical University, Yancheng, PR China; 7 Department of Radiology, The Yancheng School of Clinical Medicine of Nanjing Medical University, Yancheng, PR China

**Keywords:** Parkinson's disease, cortical thickness, surface-based morphometry, coordinate-based meta-analysis, seed-based *d* mapping

## Abstract

Parkinson’s disease (PD) is a common age-related neurodegenerative disease that affects the structural architecture of the cerebral cortex. Cortical thickness (CTh) via surface-based morphometry (SBM) analysis is a popular measure to assess brain structural alterations in the gray matter in PD. However, the results of CTh analysis in PD lack consistency and have not been systematically reviewed. We conducted a comprehensive coordinate-based meta-analysis (CBMA) of 38 CTh studies (57 comparison datasets) in 1,843 patients with PD using the latest seed-based d mapping software. Compared with 1,172 healthy controls, no significantly consistent CTh alterations were found in patients with PD, suggesting CTh as an unreliable neuroimaging marker for PD. The lack of consistent CTh alterations in PD could be ascribed to the heterogeneity in clinical populations, variations in imaging methods, and underpowered small sample sizes. These results highlight the need to control for potential confounding factors to produce robust and reproducible CTh results in PD.

## INTRODUCTION

Parkinson’s disease (PD), the second most common neurodegenerative disease after Alzheimer’s disease, affects 6.1 million individuals worldwide. The incidence of PD increases rapidly with age making it a major source of disability and global health burden [1–3]. PD is a highly clinically heterogeneous condition that is characterized by both cardinal motor symptoms, such as resting tremor, rigidity, and bradykinesia, and several non-motor symptoms throughout disease course, such as cognitive impairment, apathy, depression, anxiety, impulse control disorders, sleep disturbance, fatigue, pain, visual hallucinations, and autonomic dysfunction [4, 5]. The pathophysiology of PD is complex and involves several neural networks in addition to dopaminergic dysfunction [6–9]. Neurodegeneration may occur several years before PD is diagnosed based on characteristic motor symptoms [10]. Modern neuroimaging techniques have immensely contributed to the understanding of pathophysiology, early and differential diagnosis, severity, and progression of PD [6, 9, 11, 12].

Although degeneration of the substantia nigra is the primary histopathological feature of PD, the cerebral cortex is also affected as the disease progresses [13]. Cortical thickness (CTh) analysis of structural magnetic resonance imaging (MRI) data is a highly validated and popular surface-based technique for assessing changes in cortical gray matter (GM). Compared to voxel-based morphometry (VBM) that measures the regional GM volume as the product of the cortical surface area, CTh, and/or cortical folding, CTh analysis is more sensitive and directly assesses cortical morphology [14–17]. CTh analysis has been widely used to assess brain morphology in PD in relation to demographic and clinical characteristics, such as the age of onset, age, disease duration, motor deficits, disease stages, and divergent non-motor symptoms [17–43]. Certain study groups reported that CTh alterations served as indicators of neural degeneration in PD [15, 44], whereas others failed to identify cortical morphological features in patients with PD as compared with healthy controls (HCs) [38, 45–49]. Despite significant advances in understanding the neurobiological characteristics of PD, CTh analysis results lack consistency and have not been systematically reviewed.

A coordinate-based meta-analysis (CBMA) quantitatively combines data from individual neuroimaging studies to assess brain regions with significantly consistent structural or functional alterations in a particular neuropsychiatric disorder using the location of peak coordinates in three-dimensional (3D) anatomical spaces (x, y, z) [50, 51]. Recently, CBMA has been developed for surface-based morphometry (SBM) studies to identify consistent CTh abnormalities in major depressive disorder [52]. We conducted a CBMA of SBM studies to investigate CTh alterations in PD using seed-based *d* mapping with the permutation of subject images (SDM-PSI) [53, 54] and following the recent guidelines and recommendations [50, 51].

## RESULTS

### Included studies and characteristics

**Figure 1:**
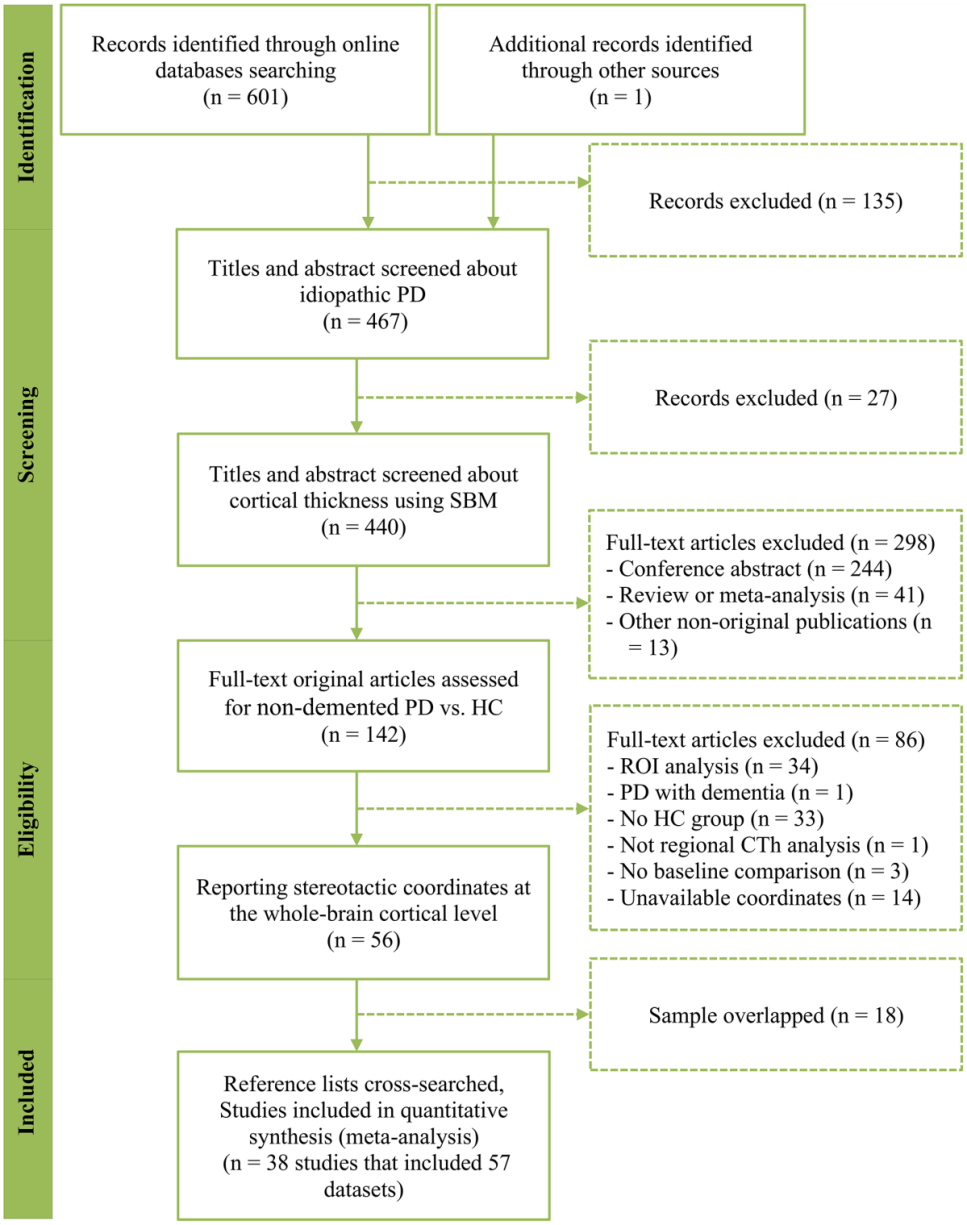
**PRISMA flow chart describing the study selection process.** PRISMA, Preferred Reporting Items of Systematic Review and Meta-Analysis; PD, Parkinson’s disease; SBM, surface-based morphometry; CTh, cortical thickness; ROI, region of interest.

A literature search produced 602 results, of which 38 studies finally met the inclusion criteria [15, 17, 20, 22, 24, 25, 31, 33–39, 41, 43–49, 55–70]. The flowchart in Figure 1 shows the study selection process. These studies included 57 PD-HC comparison datasets comprising 1,843 non-demented patients with PD (mean age: 62.87 years) and 1,172 HC subjects (mean age: 62.48 years). Sample sizes in the included datasets ranged from 8 to 151 (mean: 32.33) in the patient group and from 10 to 58 (mean: 29.3) in the HC group. The following demographic and clinical characteristics were reported in these studies: gender distribution in 53 datasets (56.32% male in the patient group and 54.48% male in the HC group), education level in 35 datasets (mean of 12.21 years in the patient group and a mean of 12.98 years in the HC group), disease duration in 54 datasets (mean: 5.43 years), Hoehn and Yahr (HY) stage in 45 datasets (mean: 2.05), United Parkinson’s disease rating scale, part III (UPDRS-III) in 52 datasets (mean: 23.62 without considering the medication state), levodopa equivalent daily dose (LEDD) in 35 datasets (mean: 506.33mg), and mini-mental state examination (MMSE) in 37 datasets (mean of 26.98 in the patient group and a mean of 28.68 in the HC group) (Supplementary Table 2). As listed in Supplementary Table 3, 3.0 Tesla MRI scanners were applied to 46 datasets (80.7%), and 50 datasets (87.7%) used thresholds corrected for multiple comparisons in the statistical analysis. Of the 57 datasets, only 26 (45.6%) underwent quality control for imaging data by a visual check and/or a subsequent manual edit as mentioned in the articles. Quality assessment scores of the included studies are listed in Supplementary Table 2.

### Main CBMA

We detected no consistent CTh differences between patients with PD and HC subjects (*n* = 57 datasets) using the threshold-free cluster enhancement (TFCE)-based family-wise error (FWE) correction (*p* < 0.05 and voxel extent ≥ 10). Although we used a less stringent significance level (uncorrected *p* < 0.005 and voxel extent ≥ 10), there was still a lack of significant results.

### Subgroup CBMA

The following datasets were used to conduct eight subgroup CBMAs: datasets obtained using 3.0 Tesla MRI scanners (*n* = 46), datasets with a slice thickness of lower than 1 mm or voxel size lower than 1×1×1 mm^3^ (*n* = 48), datasets processed using FreeSurfer software packages (*n* = 51), datasets with full width half maximum (FWHM) of the smoothing kernel size of 15 mm or less (*n* = 44), datasets with at least one covariate included in the statistical model (*n* = 42), and datasets for which thresholds were corrected for multiple comparisons (*n* = 50), datasets with quality control of imaging data (*n* = 26), and those not specifying it (*n* = 31). None of the subgroup CBMAs demonstrated significantly consistent findings using the TFCE-based FWE correction (*p* < 0.05 and voxel extent ≥ 10). Other subgroup CBMAs were not conducted because of the insufficient datasets included. Of the datasets included, only a few datasets explicitly subtyped PD patients with mild cognitive impairment (MCI) (*n* = 6). Other clinical subtypes were the case as well.

### Jackknife sensitivity, heterogeneity and publication bias analyses

Jackknife sensitivity analysis revealed that the lack of significantly consistent CTh differences between patients with PD and HC subjects survived in all combination of the datasets.

We could not perform subsequent heterogeneity and publication bias analyses because the main CBMA did not reveal any significant brain clusters.

### Meta-regression analysis

**Figure 2:**
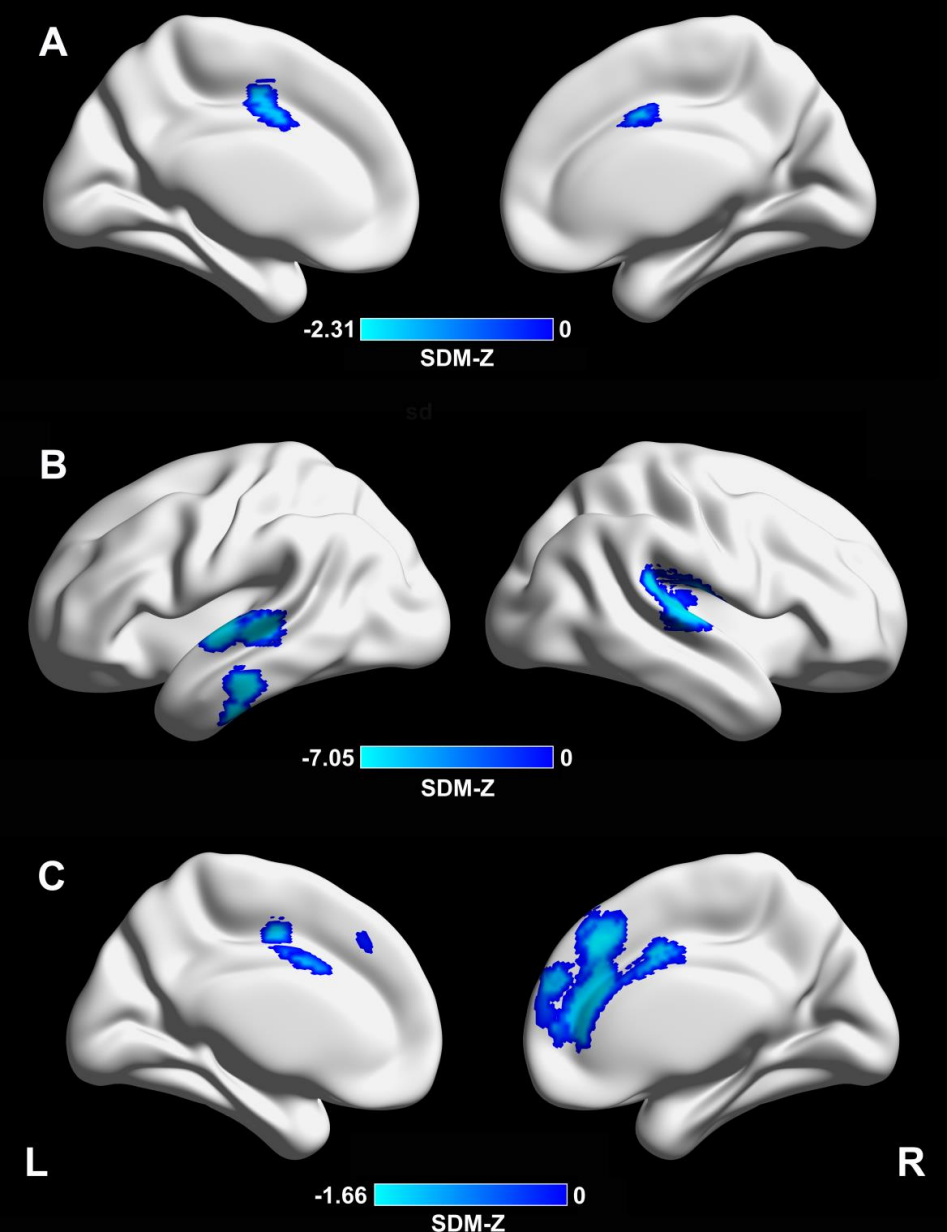
**Meta-regression analyses of clinical variables with cortical thickness.** (**A**) A longer disease duration was associated with lower CTh in the supplementary motor area/cingulate cortex (MNI coordinates: x = –4, y = –2, z = 46; BA 24; SDM-Z = –2.31; TFCE-based FWE corrected *p* = 0.009; voxels = 392). (**B**) A lower MMSE score in the PD sample was associated with lower CTh in the right superior temporal gyrus/rolandic operculum (MNI coordinates: x = 54, y = –22, z = 12; BAs 48 and 22; SDM-Z = 7.05; TFCE-based FWE corrected *p* = 0.001; voxels = 999), left superior/middle temporal gyri (MNI coordinates: x = –62, y = –12, z = 0; BAs 48, 21, and 22; SDM-Z = 6.65; TFCE-based FWE corrected *p* = 0.009; voxels = 441), and left inferior temporal gyrus (MNI coordinates: x = –60, y = –20, z = –24; BAs 20 and 21; SDM-Z = 6.57; TFCE-based FWE corrected *p* = 0.03; voxels = 169). (**C**) A higher LEDD in the PD sample was associated with lower CTh in the medial prefrontal cortex/anterior cingulate cortex (MNI coordinates: x = 4, y = 32, z = 38; BAs 32, 24, 10, and 8; SDM-Z = –1.66; TFCE-based FWE corrected *p* = 0.029; voxels = 1441). CTh, cortical thickness; MNI, Montreal Neurological Institute; BA, Brodmann area; SDM, seed-based *d* mapping; TFCE, threshold-free cluster enhancement; FWE, family-wise error; PD, Parkinson’s disease; LEDD, levodopa equivalent daily dose.

Meta-regression analysis revealed that a longer disease duration (available datasets *n* = 54) was associated with lower CTh in the supplementary motor area/cingulate cortex (Montreal Neurological Institute [MNI] coordinates: x = –4, y = –2, z = 46; Brodmann area [BA] 24; SDM-Z = –2.31; TFCE-based FWE corrected *p* = 0.009; voxels = 392, Figure 2A). In addition, a lower MMSE score in the PD sample (available datasets *n* = 37) was associated with lower CTh in the right superior temporal gyrus/rolandic operculum (MNI coordinates: x = 54, y = –22, z = 12; BAs 48 and 22; SDM-Z = 7.05; TFCE-based FWE corrected *p* = 0.001; voxels = 999), left superior/middle temporal gyri (MNI coordinates: x = –62, y = –12, z = 0; BAs 48, 21, and 22; SDM-Z = 6.65; TFCE-based FWE corrected *p* = 0.009; voxels = 441), and left inferior temporal gyrus (MNI coordinates: x = –60, y = –20, z = –24; BAs 20 and 21; SDM-Z = 6.57; TFCE-based FWE corrected *p* = 0.03; voxels = 169) (Figure 2B). A higher LEDD (available datasets *n* = 35) in the PD sample was associated with lower CTh in the medial prefrontal cortex/anterior cingulate cortex (MNI coordinates: x = 4, y = 32, z = 38; BAs 32, 24, 10, and 8; SDM-Z = –1.66; TFCE-based FWE corrected *p* = 0.029; voxels = 1441, Figure 2C). Other variables, such as mean age (available from all datasets), male percentage in the patient sample (available datasets *n* = 53), education level (available datasets *n* = 35), UPDRS-III (available datasets *n* = 52), and HY stage (available datasets *n* = 45) did not correlate with regional CTh alterations (*p* < 0.05 TFCE-based FWE corrected and cluster size ≥ 10 voxels). Results from meta-regression of UPDRS-III scores should be interpreted with caution as the medication state (on or off) was not stated in certain datasets included during the assessment of this score.

## DISCUSSION

We conducted CBMAs of SBM studies (57 datasets) to quantitatively identify consistent CTh alterations in a large pooled sample of PD patients using the latest algorithms of SDM-PSI [53, 54] and following the recent guidelines and recommendations [50, 51]. Compared with 1,172 HC subjects, we did not detect consistent CTh alterations in 1,843 patients with PD either using a TFCE-based FWE corrected or a more lenient uncorrected threshold. Furthermore, jackknife analyses and subgroup CBMAs confirmed the non-reproducibility of results. This finding is in line with the related literature that reports a widespread lack of replicability in neuroimaging [71]. Thus, CTh analysis is an unreliable neuroimaging marker in PD.

PD is a progressive neurodegenerative disorder. It has been suggested that cortical neurodegeneration begins from stage 4 (associated with early phase motor dysfunction) according to the well-established brain pathologic staging scheme for PD proposed by Braak et al. [13]. All datasets included in the current CBMA enrolled patients with PD at their symptomatic stages, i.e., showing cortical neurodegeneration that probably manifested CTh alterations, although it is still unclear whether such alterations are a direct cause of neurodegeneration or a by-product [22]. However, our CBMA did not detect consistence of CTh alterations in PD across studies. A lack of consistent CTh abnormalities in patients with PD relative to HCs may suggest that CTh analysis of MRI data may not have the power to detect such abnormalities paralleling with the neurodegeneration. Measuring the 1–5 mm thick cortex based on (usually) 1 mm^3^ voxels from MRI data is made inherently challenging; however, FreeSurfer, one of the most prominent packages for automatically estimating the brain CTh, showed good agreement with histologic measurements of CTh [72]. In addition, multiple validation studies presented good comparability for detecting CTh between advanced image processing algorithms [73]. Although reports on comparisons between histology and automated techniques from *in vivo* MRI data measuring CTh alterations in PD are lacking, we attribute the presence of inconsistent CTh abnormalities in PD to heterogeneous clinical populations, variations in imaging methods, and underpowered small sample sizes.

PD is a heterogeneous disorder in terms of clinical phenotypes (motor subtypes and variable non-motor symptoms throughout the disease course) [4, 5, 74, 75] and pathogenetic features [76, 77]. As listed in Supplementary Table 2, there were variations regarding demographic and clinical characteristics across studies. Our meta-regression analyses revealed global cognitive function, disease duration, and LEDD as confounding factors affecting CTh alterations in PD across studies. Other variables, such as age, male gender, education level, the severity of motor disability, and disease stage did correlate with regional CTh alterations. However, these results should be interpreted with caution because analyses were conducted at the study level not at an individual subject level. In individual SBM studies, different and inconsistent patterns of CTh abnormalities have been associated with demographic and clinical characteristics, such as the age of onset [18], age [19], disease duration [19–23], gender [24], the severity of motor deficits [20, 21, 25], motor laterality [26], and disease stages [20, 25, 27]. In addition, CTh abnormalities have been reported to correlate with several non-motor symptoms, such as cognitive deficits [17, 20, 23, 28–34], impulse control disorders [35, 36], depression [37–39], progressive olfactory loss [40], rapid eye movement sleep behavior disorder [41], pain [42], and mild behavioral impairment [43]. Furthermore, different subtypes of MCI in PD (amnestic MCI and non-amnestic MCI, MCI reverters and MCI non-reverters) demonstrate distinct patterns of CTh alterations [34, 58, 78]. Moreover, brain-derived neurotrophic factor (BDNF) Val66Met polymorphism in PD affects cortical thinning pattern [79]. Using a hypothesis-free, CTh data-driven approach, Uribe et al. identified three cortical phenotypes affected in non-demented PD patients [80], with different progressive patterns over time [81] that may detect prognosis markers in PD [80, 81]. Although several longitudinal studies showed that CTh alterations in PD were sensitive to time [44, 79, 81, 82], the majority of previous studies did not comprehensively distinguish PD subtypes and conducted neuroimaging analyses only at one time point. Thus, heterogeneity in clinical populations resulted in inconsistencies and a lack of reproducibility across CTh studies. Well-characterized subtype-homogeneous samples with both cross-sectional and longitudinal designs can improve the reproducibility [51].

Apart from the heterogeneity in clinical populations, inconsistent CTh alterations in PD are attributed to variations in imaging methods. As shown in Supplementary Table 3, we noted variations in scanner manufacturer and platform, field strength, head coil, MR sequence, and voxel size. MRI data of most SBM studies included in the CMBA were acquired at a single site except for the Parkinson’s Progression Markers Initiative (PPMI) cohort, a multicenter database [68]. Several studies have reported the effects of scanner platform [83, 84], field strength [85–87], pulse sequence [85, 88, 89], number of coil channels [88], scanner relocation [90], imaging sites [83, 91], type of computing workstation [92], and operating systems [92, 93] on cerebral CTh measurements. A higher field strength, multi-echo sequence, more coil channels, harmonization of CTh measurements across scanners and sites, use of homogeneous sets of platforms, and constant operating systems would reduce the bias and improve the reproducibility.

In addition, image processing-related factors might have produced inconsistent results as well. For instance, head motions in patients with PD during imaging and image artifacts induce a consistent bias in morphometric measurements [94], thus signifying the need for quality control to achieve reliable results [95, 96]. However, more than half of the studies included in our CBMA did not perform or specify a visual check and/or a secondary manual intervention for quality control. Furthermore, these studies used different processing pipelines and software packages (different versions of FreeSurfer, CIVET, and CAT12) for estimating CTh, which could have introduced bias. Similarly, a previous work reported differences in measurement between FreeSurfer version v5.0.0 and the two earlier versions (v4.3.1 and v4.5.0) [92]. Other studies demonstrated both commonalities and differences in CTh measurements between FreeSurfer and CIVET [97–99] and between FreeSurfer and CAT12 [100, 101].

With every modification and improvement in algorithms, the performance of various software packages should be evaluated [73]. Surface-based smoothing reduces noise, increases comparability across subjects, and compensates for subtle misalignments caused by image distortion [73]. The extent of smoothing applied to CTh maps critically affects the sensitivity, anatomical precision, and resolution of measurements [102]. The fact that studies included in the meta-analysis employed variable smoothing kernels in the CTh analysis could have overinterpreted the results. Thus, an optimal kernel smoothing method using a hierarchical approach based on sequential statistical thresholding was proposed [102]. As mentioned above, age and gender were associated with CTh alterations. During statistical analysis, a regression model should leave out confounding variables. However, some of the included studies did not treat them as covariates in the statistical models. In addition, an uncorrected threshold may produce spurious results in neuroimaging. Correction for multiple comparisons is essential.

A small sample size results in low statistical power, making it difficult to detect subtle differences and undermining the reliability of results [103]. Sample size estimates were heterogeneous over the cortical surface [104]. A detection of a 0.25-mm CTh difference (10% change in CTh) requires approximately 50 subjects per group [104]. Similarly, a more subtle detection of a 0.125-mm CTh difference (5% change in CTh) requires more than 250 subjects per group [104]. In line with these numbers, 47 out of the 57 datasets included in the CBMA enrolled less than 50 patients, with no dataset including more than 250 patients. The majority of these studies failed to detect CTh differences between patients with PD and HCs. Therefore, a priori power analysis should be performed to determine an appropriate sample size [105]. Furthermore, pooling multi-site large-sample datasets using standardized imaging protocols, and processing and analysis pipelines, like the PPMI, and Alzheimer's Disease Neuroimaging Initiative (ADNI), becomes increasingly important and should be highly recommended to generate reliable results.

The present CBMA had several limitations. We could not conduct additional subgroup CBMA due to the low number of studies in each PD subtype. When adequate qualified and reliable CTh studies are available for subtyping PD in the future, we may obtain more insights into the cortical characteristics relating to PD. The current evidence of lack consistency of CTh alterations in PD comes from the CBMA of cross-sectional results. Although several longitudinal studies showed the time effects of CTh alterations in PD [44, 79, 81, 82], more such studies employing large cohorts are warranted to investigate longitudinal CTh alterations to reflect neurodegenerative dynamics. CBMA is a powerful technique to quantitatively integrate neuroimaging studies; however, its algorithms are still evolving [50, 106]. Although we used SDM-PSI, the latest algorithm, the present CBMA relied only on the peak coordinate-related information reported in individual original studies. This limitation could be overcome by using image-based meta-analyses or mixed image- and coordinate-based meta-analyses [54, 106]. Further, despite being a comprehensive CBMA comprising a large pooled sample of PD, certain studies or datasets were excluded because of incomplete information reported. Data sharing and integrity of neuroimaging reports are encouraged in future studies.

### Conclusions and future perspectives

The present CBMA detected no evidence of consistent CTh alterations in patients with PD relative to HCs. This could be attributed to underpowered small sample sizes, sample heterogeneity, and variations in imaging methods. The lack of replicability across CTh studies highlights the need to control for potential confounding factors. Future studies should involve appropriate sample size, well-characterized subtype homogeneous samples, pooling of multi-site large-sample datasets, higher field strength, multi-echo sequence, more coil channels, harmonization of CTh measurements across scanners and sites, homogeneous sets of platforms, constant operating systems, quality control, well-validated algorithms, optimal smoothing kernel, appropriate covariate regression, correction for multiple comparisons, and longitudinal data to investigate CTh alterations in PD and produce robust and replicable results.

## MATERIALS AND METHODS

### Protocol and registration

The CBMA was performed following the Preferred Reporting Items of Systematic Review and Meta-Analysis (PRISMA) guidelines [107] and the recent guidelines and recommendations for neuroimaging meta-analysis [50, 51]. The protocol of this CBMA was registered at PROSPERO (http://www.crd.york.ac.uk/PROSPERO) (registration number: CRD42020148775) and published [108].

### Data sources and study selection

PubMed, Embase, and Web of Science databases were comprehensively searched using the following keywords: ((Parkinson disease) OR Parkinson*) AND ((cortical thickness) OR (cortical thinning) OR (surface-based morphometry)) for both English and non-English papers from the database inception to July 1, 2019 and updated on Feb 2, 2020. For studies published in Chinese, China National Knowledge Infrastructure (CNKI), WanFang, and SinoMed databases were also searched. Reference lists of relevant reviews and articles selected for inclusion were further manually searched.

The criteria for including articles in CBMA were: (i) articles on patients with idiopathic PD diagnosed according to the accepted criteria [109–111]; (ii) articles comparing regional CTh differences between patients with idiopathic PD and HC subjects at the whole-brain cortical level; (iii) articles reporting peak coordinates of significant clusters in standard MNI or Talairach space; and (iv) original articles published in a peer-reviewed journal in English or Chinese.

Publications were excluded if: (i) the sample size was less than seven either in the PD group or the HC group [51]; (ii) studies included PD patients with dementia; (iii) studies reported significant results without listing the three-dimensional coordinates; (iv) studies only employed regions of interest analysis, small volume corrections, or other statistical thresholds that varied depending on the brain regions; (v) studies only conducted global CTh analysis; (vi) studies lacked an HC group; (vii) studies included patient samples overlapping with that with the largest sample size; (viii) studies were longitudinal without baseline comparisons; (ix) publications were not original articles, such as conference abstracts, research protocols, letters, reviews, and editorials.

### Quality assessment

There is no objective tool to perform a quality assessment of CTh studies. Referring to a previous work [52], we used a modified 12-point checklist to assess the quality of each included study in the CBMA (Supplementary Table 1). This checklist integrated the items such as the sample characteristics, imaging-specific methodology employed, and results reported in the studies.

### Data extraction

The following data were extracted from the included studies: the first author’s family name, publication year, sample size, number of male patients, age, education (years), disease duration, UPDRS-III, HY stage, MMSE, LEDD, MR scanner manufacturer and platform, field strength, head coil, MR sequence, voxel size, imaging-processing software package, smooth kernel, statistical model, covariate, statistical threshold, peak coordinates, the height of the peaks (t-value, z-value, and p-value), and their stereotactic reference space.

L.Q.S and P.W.Z independently performed the literature search, study selection, quality assessment, and data extraction. Any inconsistencies or discrepancies were resolved by a consensus discussion.

### Data analysis

### Main CBMA

The main CBMA was conducted using the SDM-PSI software package (version 6.21, https://www.sdmproject.com/). SDM-PSI uses a new algorithm for CBMA that conducts standard voxelwise tests and, importantly, a standard PSI to assess whether effects are not null, rather than a test of convergence in other CBMA methods [53, 54]. The SDM-PSI uses standard statistical procedures to control the familywise error rate [54]. Standard procedures were followed including the preprocessing steps: conversion of peaks to a common MNI space; calculation of maps of lower and upper bounds of possible effect sizes for each study separately based on the peak information using a specific FreeSurfer GM mask [52], full anisotropy = 1, isotropic FWHM = 20mm, and voxel = 2mm; mean analysis: estimation of the most likely effect size and its standard error based on MetaNSUE algorithms [112, 113], and conducting multiple imputations and meta-analyses using a standard random-effects model and Rubin’s rules; FWE correction for multiple comparisons using common permutation tests; and use of TFCE in statistical thresholding (*p* < 0.05 and voxel extent ≥ 10). These procedures have been have been described in detail previously [53, 54] and in the SDM-PSI reference manual (https://www.sdmproject.com/manual/).

### Subgroup CBMA

Subgroup CBMA was conducted when the number of datasets was sufficient (*n* ≥ 10). Subgroup CBMA would be performed in clinical subtypes (such as PD patients with or without mild cognitive impairment) and imaging methodology variables (including datasets using 3.0 Tesla MRI scanners, slice thickness lower than 1 mm or voxel size lower than 1×1×1 mm^3^, FreeSurfer software packages, FWHM of the smoothing kernel size of 15 mm or less, at least one covariate included in the statistical model, and thresholds corrected for multiple comparisons as well as those datasets with quality control of imaging data and those not specifying it).

### Jackknife sensitivity, heterogeneity and publication bias analyses

To study the effect of each dataset on the pooled CBMA results, Jackknife sensitivity analysis was performed by iteratively repeating the same analysis K-1 times (K = the number of datasets included), discarding one dataset each time [114, 115].

For every significant cluster with a peak MNI coordinate reported in the main CBMA, information was extracted to derive standard heterogeneity statistics *I^2^* based on a standard linear hypothesis, with *I*^2^ < 50% indicating low heterogeneity. The publication bias of the significant brain cluster was assessed using the Egger test *(p* < 0.05).

### Meta-regression analysis

Meta-regression analyses were performed to study the potential effects of age, male gender, disease duration, UPDRS-III, HY stage, MMSE, and LEDD on CBMA results. Statistical significance was determined using the TFCE-based FWE corrected threshold (*p* < 0.05 and voxel extent ≥ 10).

## Supplementary Material

Supplementary Table 1

Supplementary Table 2

Supplementary Table 3
