# Supplementary material for: Cortical thickness in Parkinson's disease: a coordinate-based meta-analysis
Source: Aging (Albany NY). 2021 Jan 10;13(3):4007–23. doi: 10.18632/aging.202368 (PMC7906199; doi:10.18632/aging.202368)
Supplement: Supplementary Table 1 [file aging-13-202368-s001.pdf]

Supplementary Table 1. The checklist of quality assessment for the included cortical thickness studies.

**12-point checklist**
**Category 1: Subjects**
1. Patients were evaluated prospectively, specific diagnostic criteria were applied, and demographic data were reported.
2. Healthy comparison participants were evaluated prospectively; psychiatric and medical illnesses were excluded.
3. Important variables (e.g., age, gender, drug status, illness duration, motor symptom severity, disease stage, and cognitive function) were checked either via stratification or statistics.
4. Sample size per group: ≥ 20, scores 1; ≥ 7, scores 0.5
**Category 2: Methods for image acquisition and analysis**
5. Magnet strength: 3T, scores 1; 1.5T, scores 0.5
6. Quality control is performed.
7. The imaging technique used was clearly described so that it could be reproduced.
8. Whole brain cortical analysis was automated without a previously defined region.
9. Spatial coordinates were reported in a standard space (e.g., Talairach or MNI coordinates).
**Category 3: Results and conclusions**
10. Information about the covariates used, such as age and gender in the statistical model were provided.
11. Statistical results were corrected for multiple comparison scores 1, uncorrected scores 0.5.
12. Conclusions were consistent with the results obtained, and the limitations were discussed.
**Total score**
