# Supplementary material for: Cortical thickness in Parkinson's disease: a coordinate-based meta-analysis
Source: Aging (Albany NY). 2021 Jan 10;13(3):4007–23. doi: 10.18632/aging.202368 (PMC7906199; doi:10.18632/aging.202368)
Supplement: Supplementary Table 2 [file aging-13-202368-s002.docx]

**Supplementary Table 2. Summary of demographic and clinical characteristics of CTh studies included in the meta-analysis.**

| study | Sample sizes (male) | | Age, y | | Education, y | | Duration, y | UPDRS-III | HY stage | MMSE | | LEDD  (mg) | Quality^#^ |
| --- | --- | --- | --- | --- | --- | --- | --- | --- | --- | --- | --- | --- | --- |
|  | **PD** | **HCs** | **PD** | **HCs** | **PD** | **HCs** |  |  |  | **PD** | **HCs** |  |  |
| Acosta-Cabronero et al. (2016) | 25 (20) | 50 (28) | 63.6 | 63.6 | NA | NA | 6 | 16.3 | 2.2 | 26.7 | 29.0 | 748 | 9.5 |
| Biundo et al. (2015) | 52 (32)^a^  58 (38)^b^ | 33 (13) | 63.1  60.3 | 55.3 | 11.3  10.9 | 11.6 | 8.0  9.0 | 28.5  26.7 | 2.3  2.4 | 27  26.4 | 28.2 | 722.6  923.1 | 11.5  11.5 |
| Carriere et al. (2014) | 10 (6)^c^  10 (6)^d^ | 10 (4) | 60.7  67.2 | 66.8 | 12.5  10.0 | 10.8 | 11.9  11.9 | 28.7  28.1 | NA  NA | NA^&^  NA^&^ | NA^&^ | 1072.1  857.2 | 9.0  9.0 |
| Carriere et al. (2015) | 17 (13)^a^  19 (15)^b^ | 16 (12) | 57.4  57.4 | 56 | NA  NA | NA | 7.2  6.9 | 19.6  20.5 | 2.3  2.4 | 28.9  28.4 | 28.6 | 817  908 | 9.0  9.0 |
| Cerasa et al. (2013) | 29 (15)^e^  30 (17)^f^ | 24 (13) | 60.1  62.1 | 63.6 | NA  NA | NA | 6  7.3 | 23  20 | 2.3  2.5 | 27.3  27 | 28 | 520.6  534 | 10.5  10.5 |
| Chung et al. (2019) | 50 (20)^g1^ | 30 (11) | 69.52 | 70.63 | 8.83 | 10.28 | 2.58 | 25.53 | NA | 26.00 | 28.33 | NA | 10.5 |
| Danti et al. (2015) | 18 (15)^h^  18 (14)^g2^ | 18 (12) | 60.6  66.5 | 62.3 | 9.9  9.7 | 9.6 | 1.5  1.67 | 10.7  6.4 | 1.3  1.6 | 28.7  26.4 | NA | NA | 10.5  10.5 |
| Deng et al. (2016) | 67 (37) | 35 (24) | 65.31 | 67.3 | 11.7 | 12.74 | 8.1 | 34.11 | 2.7 | 15.00 | 29.5 | 568.97 | 11.0 |
| Gao et al. (2018) | 28 (17)^i^  32 (12)^j^ | 29 (12) | 57.32  61.59 | 59.17 | 10.04  9.81 | 9.69 | 2.34  2.54 | 19.29  31.08 | 1.5  1.5 | 28.14  27.25 | NA | NA  NA | 9.0  9.0 |
| Garcia-Diaz et al. (2014) | 36 (28) | 20 (11) | 64.37 | 69.15 | 12.39 | 11.35 | 10.342 | 17.53 | 2.07 | > 25 | NA | 767.63 | 10.5 |
| Garcia-Diaz et al. (2018) | 92 (37) | 36 (19) | 64 | 63.4 | 10.6 | 11.4 | 8.4 | 16.4 | 1.9 | > 25 | NA | 803.7 | 10.5 |
| Gasca-Salas et al. (2019) | 15 (9)^g3^  8 (6)^g4^ | 18 (11) | 70.1  69.6 | 67.6 | < 8: 13, ≥ 8: 5  < 8: 5, ≥ 8: 3 | < 8: 13, ≥ 8: 5 | 13.3  12.2 | 17.8 (on)  14.6 (on) | 2.9  2.38 | 26.1  26.7 | NA | 1198.1  961.2 | 9.5  9.5 |
| Gerrits et al. (2016) | 93 (61) | 45 (27) | 62.8 | 60.6 | **NA*** | **NA*** | **3.0** | 25.5 | 2 | 27.7 | 27.8 | 509 | 12 |
| Guimaraes et al. (2016) | 16 (NA)^i^  21 (NA)^j^  11 (NA)^k^ | 40 (NA) | 59.31  58.5  62.07 | 57.60 | 8.05  7.05  4.84 | NA | 2.5  7  12.14 | 10.88  16.2  28.35 | 1.25  2.57  4.42 | NA^$^  NA^$^  NA^$^ | NA^$^ | NA | 10.0  10.5  10.0 |
| Huang et al. (2016) | 34 (17) | 45 (24) | 59.25 | 57.0 | NA | NA | 3.95 | 42.3 | 2.5 | 26.15 | NA | NA | 11.5 |
| Ibarretxe-Bilbao (2012) | 16 (NA) | 15 (NA) | 56.13 | 57.58 | 10.96 | 13 | 3.06 | 15.44 | 1.81 | 29.56 | 29.87 | 282.50 | 9.0 |
| Jubault et al. (2011) | 49 (33) | 33 (17) | 63.3 | 65.0 | 14.2 | 14.3 | 4.4 | 28.7 | 1-2.5 | NA^@^ | NA^@^ | 411 | 11.0 |
| Kamagata et al. (2017) | 30 (12) | 28 (10) | 67.6 | 66.5 | NA | NA | 6.4 | 16.1 | 2.1 | NA | NA | NA | 11.5 |
| Kunst et al. (2019) | 23 (17)^h^  24 (16)^g2^ | 58 (18) | 61  65.1 | 67.5 | 16.2  14 | 15.4 | NA  NA | NA  NA | NA  NA | 28.2  27 | 28.5 | 815.5  909 | 11.5  11.5 |
| Luo et al. (2016) | 56 (28) | 56 (28) | 52.28 | 52.52 | NA | NA | 2.1 | 28.02 | 1.96 | 27.21 | NA | 0 | 11.5 |
| Lyoo et al. (2010) | 48 (22) | 56 (28) | 60.2 | 60.5 | NA | NA | 1.97 | 20.0 | 1.9 | 28.3 | NA | 0 | 11.0 |
| Madhyastha et al. (2015) | 23 (16) | 21 (9) | 63.96 | 61.90 | 16.41 | 15.9 | 6.55 | 22.52 | 2.04 | NA^@^ | NA^@^ | NA | 11.5 |
| Mak et al. (2015) | 66 (41)^h^  39 (29)^g2^ | 37 (21) | 62.9  69.4 | 65.7 | 13.8  11.6 | 13.9 | 2.02  2.08 | 25.3  29 | 1.9  2.1 | 29.1  28.1 | 29.4 | 143.1  248.7 | 12.0  12.0 |
| Nurnberger et al. (2017) | 13 (7) | 18 (9) | 62.6 | 64.4 | NA | NA | 4.5 | 16.8 | 1.2 | NA | NA | NA | 9.0 |
| Pagonabarraga et al. (2013) | 26 (14)^h^ | 18 (10) | 71.5 | 68.2 | 9.0 | 10.4 | 7.3 | 24 | 2.2 | NA^&^ | NA^&^ | 791 | 11.5 |
| Pereira et al. (2012) | 20 (13) | 20 (14) | 64 | 59.1 | NA | NA | 6.8 | 24.9 | 2.4 | 28.5 | 29.8 | 627 | 10.5 |
| Pereira et al. (2019) | 151 (94) | 31 (20) | 60.6 | 58.5 | 15.4 | 16.5 | NA | NA^§^ | 1.6 | NA^@^ | NA^@^ | 0 | 10.5 |
| Rahayel et al. (2019) | 15 (5)^l^  15 (10)^m^ | 41 (25) | 63.1  66.7 | 63.3 | 15.7  14.2 | 14.6 | 3.7  3.9 | 17.6  24.1 | NA  NA | NA  NA | NA | 447.8  625.2 | 10.0  10.0 |
| Tessitore et al. (2016) | 15 (12)^a^  15 (13)^b^ | 24 (17) | 63.14  62.87 | 63.54 | 12.9  9.8 | 10.3 | 6.6  5.3 | 12.1  10.9 | 1.4  1.3 | NA  NA | NA | 532.1  477.3 | 10.0  10.0 |
| Wilson et al. (2019) | 27 (15)^n^  27 (14)^o^  26 (12)^p^ | 30 (16) | 57.2  60.7  60.1 | 60.2 | NA  NA  NA | NA | 3.4  7.2  13.3 | 20.6  32.1  49.1 | 1.7  2.4  3.4 | 29.6  29.6  29.6 | 29.8 | NA  NA  NA | 10.5  10.5  10.5 |
| Worker et al. (2014) | 14 (7) | 19 (10) | 64.6 | 63.8 | NA | NA | 6.6 | 21.8 | 2.5 | 29.5 | NA | NA | 9.5 |
| Xiang et al. (2019) | 21 (13)^q^  22 (13)^r^ | 16 (5)  20 (10) | 46.52  60.82 | 48.00  62.65 | 6.76  7.82 | 8.19  7.65 | 2.25  1.83 | 26.71  21.09 | 1.71  1.36 | 27.05  26.82 | 27.88  26.1 | NA  NA | 11.5  11.5 |
| Yadav et al. (2016) | 43 (43)^s^  21 (0)^t^ | 12 (12)  34 (34) | 71.2  69.5 | 73.0  69.3 | NA  NA | NA  NA | 3.67  3.69 | NA  NA | NA  NA | 27.5  27.8 | 29.0  29.2 | NA  NA | 11.0  11.0 |
| Yao et al. (2014) | 12 (4)^u^  12 (3)^v^ | 14 (6) | 63.4  67.6 | 64.1 | NA  NA | NA | 8.4  10.0 | 18.0  20.9 | 2.8  3.2 | 28.5  27.6 | 29.1 | 704.9  978.7 | 9.0  9.0 |
| Yoo et al. (2015) | 43 (25)^w^ | 23 (NA) | 67.1 | 71 | 9.0 | NA | 3.0 | 26.8 | NA | 28.0 | 28.0 | 530.0 | 10.5 |
| Yoon et al. (2019) | 40 (27)^x^  20 (15)^y^ | 29 (14) | 70.2  71.3 | 68.7 | 15.4  14.4 | 16.7 | 5.3  6.4 | 16.7  22.0 | NA  NA | NA^@^  NA^@^ | NA^@^ | 730.9  910.7 | 10.5  10.5 |
| Zanigni et al. (2016) | 11 (8) | 22 (12) | 58.0 | 56.4 | 13.0 | NA | 1.9 | 13.0 | 1.55 | 29.0 | NA | NA | 9.5 |
| Zhang et al. (2015) | 71 (40) | 48 (30) | 62.21 | 61.70 | 14.97 | 15.78 | 4.7 | 21.4 | 1.7 | NA | NA | NA | 10 |

CTh, cortical thickness; PD, Parkinson’s disease; HCs, healthy controls; y, years; UPDRS-III, Unified Parkinson’s Disease Rating Scale, part III; HY, Hoehn and Yahr scale; MMSE, mini-mental state examination; LEDD, Levodopa equivalent daily dose; NA, not available; a, Parkinson’s disease patients without impulse control disorders; b, Parkinson’s disease patients with impulse control disorders; c, Parkinson’s disease patients without apathy; d, Parkinson’s disease patients with apathy; e, non-dyskinetic Parkinson’s disease patients; f, dyskinetic Parkinson’s disease patients; g1, Parkinson’s disease patients with non-amnestic mild cognitive impairment; g2, Parkinson’s disease patients with mild cognitive impairment; g3, Parkinson’s disease patients with mild cognitive impairment non-converters; g4, Parkinson’s disease patients with mild cognitive impairment converters; h, Parkinson’s disease patients without mild cognitive impairment; i, mild Parkinson’s disease patients (HY 1–1.5); j, moderate Parkinson’s disease patients (HY 2–2.5); k, severe Parkinson’s disease patients (HY 3–5); l, Parkinson’s disease with rapid eye movement sleep behavior disorder; m, Parkinson’s disease with rapid eye movement sleep behavior disorder; n, mild Parkinson’s disease patients (HY stage 1–2), o, moderate Parkinson’s disease patients (HY 2–3); p, severe Parkinson’s disease patients (HY 3–4); q, early onset Parkinson’s disease; r, late onset Parkinson’s disease; s, male patients with Parkinson’s disease; t, female patients with Parkinson’s disease; u, Parkinson’s disease without visual hallucination; v, Parkinson’s disease with visual hallucination; w, Parkinson’s disease patients without punding behavior; x, Parkinson’s disease without mild behavioral impairment; y, Parkinson’s disease with mild behavioral impairment. &, Mattis Dementia Rating Scale; $, Scales for Outcomes in Parkinson’s Disease-Cognition; @, Montreal Cognitive Assessment; §, MDS-UPDRS-III; * Education level was determined by using a Dutch education system.
