# Supplementary material for: Cortical thickness in Parkinson's disease: a coordinate-based meta-analysis
Source: Aging (Albany NY). 2021 Jan 10;13(3):4007–23. doi: 10.18632/aging.202368 (PMC7906199; doi:10.18632/aging.202368)
Supplement: Supplementary Table 3 [file aging-13-202368-s003.docx]

**Supplementary Table 3. Imaging characteristics of the CTh studies included in the meta-analysis.**

| Study | MRI scanner | Field strength | Head coil | MRI sequence | Voxel size  (mm^3^) | Software | FWHM (mm) | Cross-sectional analysis | Covariate | Threshold | Quality control |
| --- | --- | --- | --- | --- | --- | --- | --- | --- | --- | --- | --- |
| Acosta-Cabronero et al. (2006) | Verio, Siemens | 3.0 Tesla | 32-channel | MPRAGE | 1*1*1 | FreeSurfer v5.3.0 | default | default | NA | P < 0.05 (MCS) | NA |
| Biundo (2015) | Achieva, Philips | 1.5 Tesla | 8-channel | TFE | 0.87 (slice thickness) | FreeSurfer v5.1 | 15 | Vertex-wise GLM | age, MMSE | P < 0.05 (FDR) | Yes |
| Carriere (2014) | Achieva, Philips | 3.0 Tesla | 8-channel | MPRAGE | 0.750*0.727*0.727 | FreeSurfer v5.0 | 20 | Vertex-wise GLM | NA | P < 0.05 (RFT) | NA |
| Carriere (2015) | Achieva, Philips | 3.0 Tesla | 8-channel | MPRAGE | 0.750*0.727*0.727 | FreeSurfer v5.0 | 6 | Vertex-wise GLM | NA | P < 0.05 (RFT) | NA |
| Cerasa (2013) | Signa NV/I, GE | 1.5 Tesla | NA | SPGR | 1.2 (slice thickness) | FreeSurfer | 10 | Vertex-wise GLM | age, gender | P < 0.05 (NA) | Yes |
| Chung (2019) | Achieva, Philips | 3.0 Tesla | SENSE factor = 2 | TFE | 0.859*0.859 *1 | FreeSurfer | 20 | Vertex-wise ANCOVA | age, gender, education, ICV | P < 0.05 (RFT) | NA |
| Danti (2015) | Symphony, Siemens | 1.5 Tesla | quadrature birdcage | MPRAGE | 1 (slice thickness) | FreeSurfer 5.3.0 | 5 | Vertex-wise GLM | age, gender | P < 0.05 (MCS) | Yes |
| Deng (2016) | Tim Trio, Siemens | 3.0 Tesla | 8-channel | MPRAGE | 1*1*1 | CIVET | 20 | vertex-wise ANCOVA | brain size, cortical surface | P < 0.05 (RFT) | NA |
| Gao (2018) | GE | 3.0 Tesla | NA | SPGR | 0.469*0.469*1.0 | FreeSurfer  v5.3.0 | NA | Vertex-wise GLM | NA | P < 0.001 (NA) | NA |
| Garcia-Diaz (2014) | Magnetom Trio, Siemens | 3.0 Tesla | NA | NA | 1*1*1 | FreeSurfer v5.1 | 15 | Vertex-wise GLM | NA | P < 0.05 (MCS) | Yes |
| Garcia-Diaz (2018) | Magnetom Trio, Siemens | 3.0 Tesla | NA | NA | 1*1*1 | FreeSurfer v5.1 | 15 | Vertex-wise GLM | NA | P < 0.05 (MCS) | Yes |
| Gasca-Salas (2019) | Magnetom SP, Siemens | 1.5 Tesla | NA | NA | 0.977*1.5*0.977 | FreeSurfer v5.1 | 20 | Vertex-wise GLM | age, gender | P < 0.05 (FDR) | NA |
| Gerrits (2016) | Signa HDxt, GE | 3.0 Tesla | 8-channel | gradient-echo | 1* 0.98*0.98 | FreeSurfer v5.3 | 10 | Vertex-wise GLM | total GM volume | P < 0.05 (MCS) | Yes |
| Guimaraes (2016) | Achieva, Philips | 3.0 Tesla | NA | NA | 1*1*1 | CIVET v1.1.10 | NA | Vertex-wise GLM | age, gender | P < 0.05 (RFT) | NA |
| Huang (2016) | Signa, GE | 3.0 Tesla | NA | FSPGR | 1.2 (slice thickness) | FreeSurfer v5.3 | NA | Vertex-wise GLM | age, gender | P < 0.05 (MCS) | Yes |
| Ibarretxe-Bilbao (2012) | Tim Trio, Siemens | 3.0 Tesla | NA | MPRAGE | 1*1*1 | FreeSurfer v5.1 | 15 | Vertex-wise GLM | NA | P < 0.05 (MCS) | NA |
| Jubault (2011) | Tim, Siemens | 3.0 Tesla | 12-channel | gradient-echo | 1*1*1 | CIVET | 20 | Vertex-wise GLM | age, gender | P < 0.05 (RFT) | NA |
| Kamagata (2017) | Achieva, Philips | 3.0 Tesla | NA | MPRAGE | 0.86 (slice thickness) | FreeSurfer v5.0.0 | 15 | Vertex-wise GLM | age, gender, eTIV | P < 0.05 (MCS) | Yes |
| Kunst (2019) | Prisma, Siemens | 3.0 Tesla | NA | MPRAGE | 1 (slice thickness) | FreeSurfer v6.0 | 10 | Vertex-wise GLM | age, gender, education, GDS scores | P < 0.05 (MCS) | Yes |
| Luo (2016) | Excite, GE | 3.0 Tesla | 8-channel | SPGR | 0.47*0.47*1 | FreeSurfer v5.3.0 | 15 | Vertex-wise GLM | age, gender | P < 0.05 (MCS) | Yes |
| Lyoo (2010) | Signa Excite, GE | 3.0 Tesla | NA | SPGR | 1*1*1 | Freesurfer v4.3.0 | NA | Vertex-wise GLM | age, gender | P < 0.001 (Uncorr) | Yes |
| Madhyastha (2015) | Achieva, Philips | 3.0 Tesla | 32-channel | MPRAGE | 1*1*1 | FreeSurfer v5.3.0 | 15 | Vertex-wise multilevel model | gender | P < 0.05 (MCS) | Yes |
| Mak (2015) | Achieva, Philips | 3.0 Tesla | NA | MPRAGE | 1.15*1.15*1.2 | FreeSurfer v5.3 | 15 | Vertex-wise GLM | age, gender, education | P < 0.05 (MCS) | Yes |
| Nurnberger (2017) | Trio, Siemens | 3.0 Tesla | 8-channel | gradient-echo | 1*1*1 | FreeSurfer v5.3 | 5 | NA | NA | P < 0.01 (MCS) | NA |
| Pagonabarraga (2013) | Achieva, Philips | 3.0 Tesla | NA | MPRAGE | 0.889*0.889*1.2 | FreeSurfer v4.3.1 | 10 | Vertex-wise GLM | age and education | P < 0.01 (Uncorr) | Yes |
| Pereira (2012) | Trio Tim Siemens | 3.0 Tesla | NA | MPRAGE | 1*1*1 | FreeSurfer v4.3.1 | 15 | Vertex-wise GLM | age, gender, ICV | P < 0.05 (MCS) | NA |
| Pereira (2019) | Trio Tim Siemens | 3.0 Tesla | NA | MPRAGE | 1*1*1 | FreeSurfer v6.0 | 15 | Vertex-wise GLM | age, gender, education | P < 0.05 (MCS) | NA |
| Rahayel (2019) | Trio Tim Siemens | 3.0 Tesla | 12-channel | MPRAGE | 1*1*1 | CAT12 | 15 | GLM | age, gender, education | P < 0.05 (TFCE) | NA |
| Tessitore (2016) | GE | 3.0 Tesla | 8-channel | IR-FSPGR | 1*1*1.2 | FreeSurfer v4.5 | 10 | Vertex-wise GLM | age, gender | P < 0.05 (FDR) | NA |
| Wilson (2019) | Siemens | 3.0 Tesla | NA | MPRAGE | 1*1*1 | FreeSurfer v5.3.0 | 10 | Vertex-wise GLM | NA | P < 0.05 (MCS) | Yes |
| Worker (2014) | Signa LX NV/I, GE | 1.5 Tesla | quadrature  birdcage | IR-SPGR | 0.9375*0.9375*1.5 | FreeSurfer v5.1.0 | 10 | Vertex-wise GLM | age, gender | P < 0.05 (MCS) | NA |
| Xiang (2019) | Siemens | 3.0 Tesla | 16-channel | MPRAGE | 1 (slice thickness) | FreeSurfer v5.3.0 | 15 | Vertex-wise GLM | age, gender | P < 0.05 (MCS) | Yes |
| Yadav (2016) | Sonata, Siemens | 1.5 Tesla | vendor-supplied | MPRAGE | 1.2 (slice thickness) | FreeSurfer v5.3.0 | 15 | Vertex-wise GLM | age, gender | P < 0.05 (FDR) | Yes |
| Yao (2014) | Achieva, Philips | 3.0 Tesla | 8-channel | MPRAGE | 1*1*1 | FreeSurfer v4.3.1 | 15 | Vertex-wise GLM | NA | P < 0.05 (MCS) | NA |
| Yoo (2015) | Intera, Philips | 3.0 Tesla | SENSE factor = 2 | TFE | 0.98*0.98*1.2mm | NA | NA | t-statistics | age, sex, education | P < 0.05 (RFT) | NA |
| Yoon (2019) | DISCOVERY MR750, GE | 3.0 Tesla | NA | IR-FSPGR | 1*1*1 | FreeSurfer v6.0.0 | 10 | Vertex-wise GLM | age, education | P < 0.05 (MCS) | NA |
| Zanigni (2016) | Signa HDx, GE | 1.5 Tesla | birdcage | FSPGR | 1*1*1 | FreeSurfer v4.4.0 | 10 | Vertex-wise GLM | age, sex, TIV | P < 0.001 (Uncorr) | NA |
| Zhang (2015) | Trio Tim, Siemens | 3.0 Tesla | 8-channel | MPRAGE | 1*1*1 | FreeSurfer v5.3 | 10 | hierarchical Bayesian model | age | posterior probability > 0.9 (MCMC) | NA |

CTh, cortical thickness; MRI, magnetic resonance imaging; FWHM, full width half maximum; MPRAGE, Magnetization Prepared Rapid Gradient Echo; NA not available; MCS Monte Carlo simulations; TFE, Turbo Field Echo; GLM, general linear model; MMSE, Mini-Mental State Examination; FDR, false discovery rate; RFT, random field theory; SPGR, spoiled gradient echo; ANCOVA, analysis of covariance; ICV, intracranial volume; GM, gray matter; FSPGR, fast spoiled gradient recalled echo sequence; eTIV, estimated total intracranial volume; GDS geriatric depression scale; Uncorr, uncorrected; IR-FSPGR, inversion recovery prepared fast spoiled gradient recalled sequence; TFCE, threshold-free cluster enhancement; MCMC, Markov Chain Monte Carlo
